# Development of neural specialization for print: Evidence for predictive coding in visual word recognition

**DOI:** 10.1371/journal.pbio.3000474

**Published:** 2019-10-10

**Authors:** Jing Zhao, Urs Maurer, Sheng He, Xuchu Weng

**Affiliations:** 1 Institutes of Psychological Sciences, Hangzhou Normal University, Hangzhou, China; 2 Zhejiang Key Laboratory for Research in Assessment of Cognitive Impairments, Hangzhou, China; 3 Department of Psychology, Chinese University of Hong Kong, Hong Kong, China; 4 Brain and Mind Institute, Chinese University of Hong Kong, Hong Kong, China; 5 State Key Laboratory of Brain and Cognitive Science, Institute of Biophysics, Chinese Academy of Sciences, Beijing, China; 6 Department of Psychology, University of Minnesota, Minneapolis, Minnesota, United States of America; 7 Institute for Brain Research and Rehabilitation, South China Normal University, Guangzhou, China; New York University, UNITED STATES

## Abstract

How a child’s brain develops specialization for print is poorly understood. One longstanding account is selective neuronal tuning to regularity of visual-orthographic features, which predicts a monotonically increased neural activation for inputs with higher regularity during development. However, we observed a robust interaction between a stimulus’ orthographic regularity (bottom-up input) and children’s lexical classification ability (top-down prediction): N1 response, which is the first negative component of the event-related potential (ERP) occurring at posterior electrodes, was stronger to lower-regularity stimuli, but only in children who were less efficient in lexically classifying these stimuli (high prediction error). In contrast, N1 responses were reduced to lower-regularity stimuli in children who showed high efficiency of lexical classification (low prediction error). The modulation of children’s lexical classification efficiency on their neural responses to orthographic stimuli supports the predictive coding account of neural processes of reading.

## Abstract 2

How a child’s brain develops specialization for print is poorly understood. A study of children learning to read Chinese shows that neural specialization for print develops through and is characterized by interactions between regularities of visual inputs and efficiency of top-down predictions.

## Introduction

Through extensive learning, humans gain the ability to quickly recognize visual patterns in the environment, which is important for acquiring cultural skills such as reading. The ability to efficiently recognize visual words relies on neural specialization for print [1]. One further fundamental issue is how a child’s brain develops specialized visual-orthographic processing. One longstanding theoretical account, referred to as feature detection model, proposes that selective tuning to visual-orthographic features, as a result of detecting bottom-up stimulus features that are repeatedly encountered in the course of children’s learning to read, serves as the main mechanism (see Local Combination Detector model or Orthographic Tuning Hypothesis) [2,3]. On the contrary, the predictive coding model claims that the specialization emerges and develops through a predictive coding mechanism by which visual-orthographic features (bottom-up information) interact early on with lexical classification (top-down predictions), which becomes more efficient as reading experience increases (see Interactive Account) [4]. Therefore, the 2 models, in principle, make distinct theoretical hypotheses about developmental profiles of the effect of visual-orthographic regularity on early neural activation. In the feature detection model, stimuli detected as having higher regularity would generate stronger neural activation, and during development, such a monotonic dependence of neural response on stimulus regularity would become more evident as children become more skilled in detecting the orthographic regularity of stimuli. By contrast, the predictive coding model predicts less neural activation to stimuli with higher regularity as they are predicted more efficiently due to low prediction errors, and a non-monotonic neural response to stimuli with different orthographic regularities during development: at the beginning of learning to read, the neural activation is low as there is little basis for top-down prediction; during learning, the neural activation would become stronger for lower-regularity stimuli because they are recognized as word-like, thus engaging top-down prediction with a large prediction error; with more learning and more efficient lexical classification, stimuli not conforming to orthographic regularity would be more efficiently rejected and less likely to engage the top-down prediction, leading to decreased neural activation.

However, conclusive empirical evidence adjudicating between these rival models is lacking, although there has been a considerable body of research on these two models. The non-monotonic neural response is assumed in the predictive coding model, as stimuli with low orthographic regularity (e.g., symbol strings in [5,6]) may not engage word-related predictions [4], leading to similar activation profiles during learning to read in both predictive coding and feature detection models. Therefore, distinct theoretical hypotheses can only be made for developmental profiles of neural responses to stimuli that look more word-like than symbol strings and with different degrees of visual-orthographic regularity (e.g., words and pseudowords, or pseudowords and irregular letter strings in alphabetic languages, or real characters and pseudo characters, or pseudo characters and false characters in Chinese).

To test the two theoretical models and further explore the mechanism underlying the development of the neural specialization for print, we characterized the developmental profile of neural responses to stimuli with different degrees of visual-orthographic regularity in children at different stages of learning to read. We found a non-monotonic pattern of the neural responses over the course of development: at the beginning, no difference was found among neural responses to stimuli with different degrees of visual-orthographic regularity; during learning, neural response was stronger to stimuli with lower regularity when children were less efficient in lexically classifying these stimuli; and with more learning, neural responses were reduced to lower-regularity stimuli when children showed higher efficiency of lexical classification, in agreement with predictive coding but not feature detection models.

## Results

### Behavior

**Fig 1:**
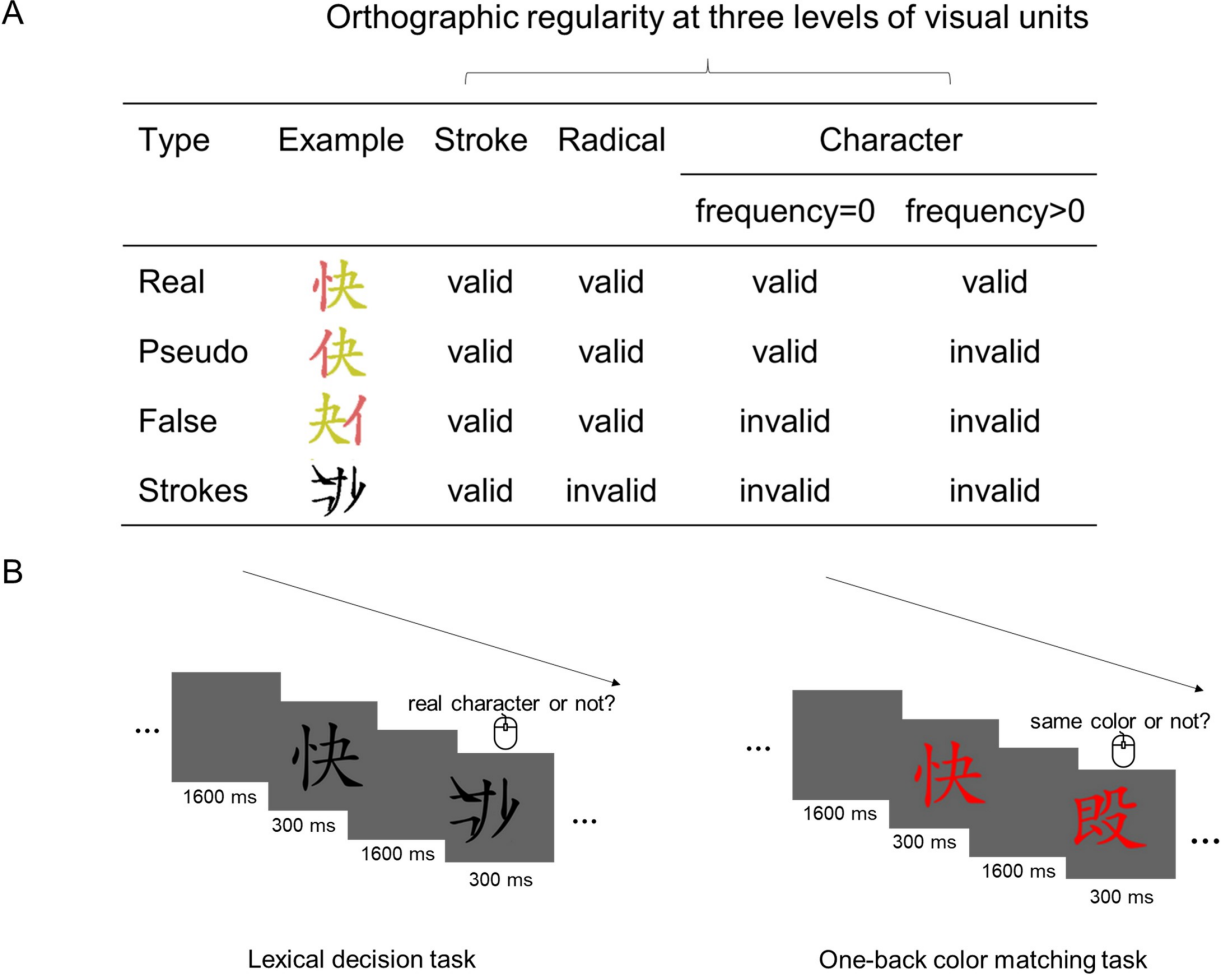
Design in the behavioral and ERP experiments. (A) Examples of stimuli with decreasing visual-orthographic regularity for adult skilled readers in Chinese script: real, pseudo, false characters, and stroke combination. These stimuli are valid at different levels of visual units (i.e., stroke, radical, and character). Stroke combinations were constructed by combining strokes of characters that do not form valid radicals (invalid at radical level). False characters consisted of valid radicals, but they were placed in illegal positions and did not form valid characters (invalid at character level). Pseudo characters consisted of radicals at orthographic legal positions, but the resulting “characters” do not exist in the lexicon (frequency = 0). Real characters were valid at all levels of visual units. The two different colors in the examples are for demonstration only to show the two radicals. (B) Schematic depiction of the lexical decision task in the behavioral experiment (left panel) and one-back color matching task in the ERP experiment (right panel). In the lexical decision task, children were instructed to judge as accurately and as fast as possible whether a stimulus was a real Chinese character or not. In the one-back color matching task, each stimulus was presented in one of 3 colors (red, green, or yellow). Children were asked to press a button as accurately and as fast as possible whenever the same color occurred twice in a row. ERP, event-related potential.

The behavioral experiment investigated the development of perceived orthographic regularity and used it as a measure of efficiency of children’s lexical classification of characters and other stimuli during learning to read. Four types of stimuli, which had different degrees of visual-orthographic regularity for adult skilled Chinese readers, were used: real characters, pseudo characters, false characters, and stroke combinations (see Fig 1A). A lexical decision task was adopted, in which children were instructed to judge whether a stimulus was a real Chinese character or not (Fig 1B, left panel). With the 4 types of stimuli and the lexical decision task, we could estimate the extent to which a certain stimulus type was perceived as having regular orthography and the efficiency with which this stimulus type could be classified as a real character. When participants responded ‘character’ to a stimulus, this stimulus would be perceived as a real character. The range of the percentage of ‘character’ response is 0% to 100%. In the framework of these 2 models, the minimum (0%) means that all stimuli in this group were rejected as real characters, suggesting that participants perceive that the orthography of these stimuli is mostly irregular and there was no drive for word-related prediction (and have minimum prediction error). The maximum (100%) means that all stimuli are recognized as real characters, suggesting that participants perceive the orthography of these stimuli as mostly regular and predict these stimuli efficiently (and have low prediction error). The chance level (50%) response means that participants are mostly uncertain about whether the stimuli are real characters or not, suggesting that participants are more uncertain about the stimuli’s orthographic regularity and predict these stimuli more inefficiently (and have higher prediction errors).

**Fig 2:**
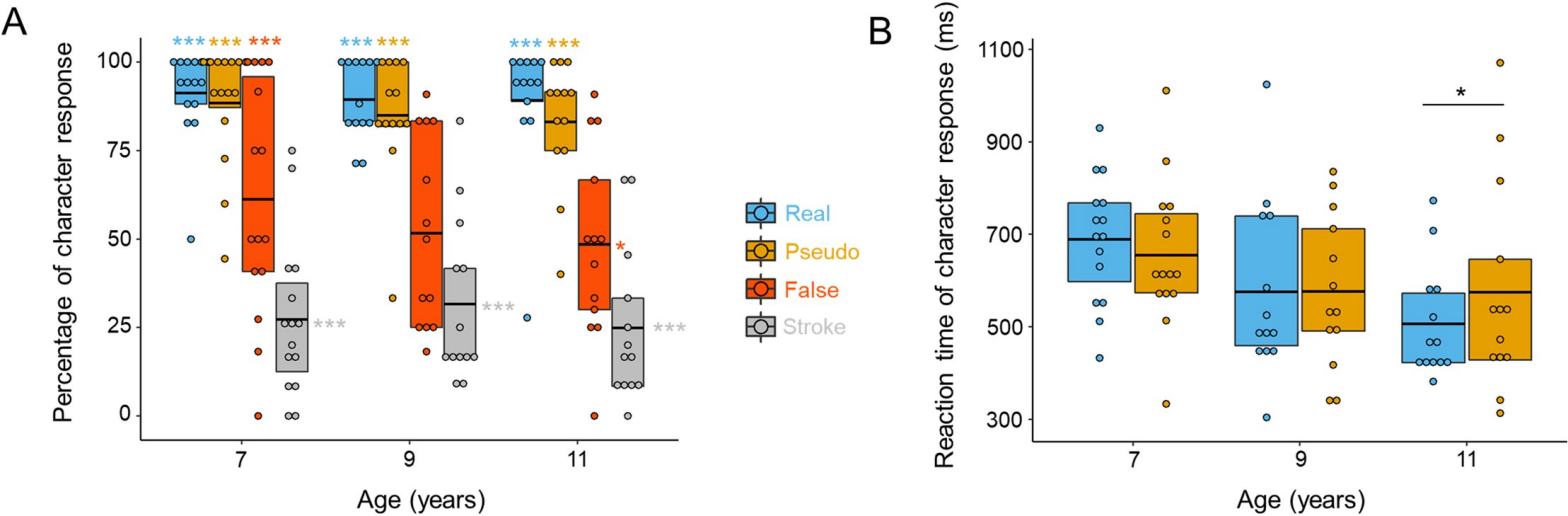
Development of orthographic regularity perception and lexical classification efficiency for 4 types of stimuli. (A) Percentage of character response. Hit for real characters and false alarm for the other 3 stimulus types in the behavioral lexical decision task. The significance tests were two sided, and they were binominal tests against chance level (50%). The percentages of character response to real and pseudo characters were high above chance level; the percentage of character response to stroke combinations was well below chance level at 3 ages. Importantly, 7-year-olds showed a higher percentage of character response to false characters than chance level. The percentage of character response to false characters was closest to chance in 9-year-olds. With more reading experience, the percentage of character response to false characters was lower than chance level. (B) Reaction time data of character responses; 7- and 9-year-olds showed no reaction time differences between pseudo and real characters, while 11-year-olds showed longer reaction times when classifying pseudo characters as real characters. Lines in the boxes denote means; **p < 0.01, *p < 0.05. See 10.6084/m9.figshare.8948912 for subject data.

**Fig 3:**
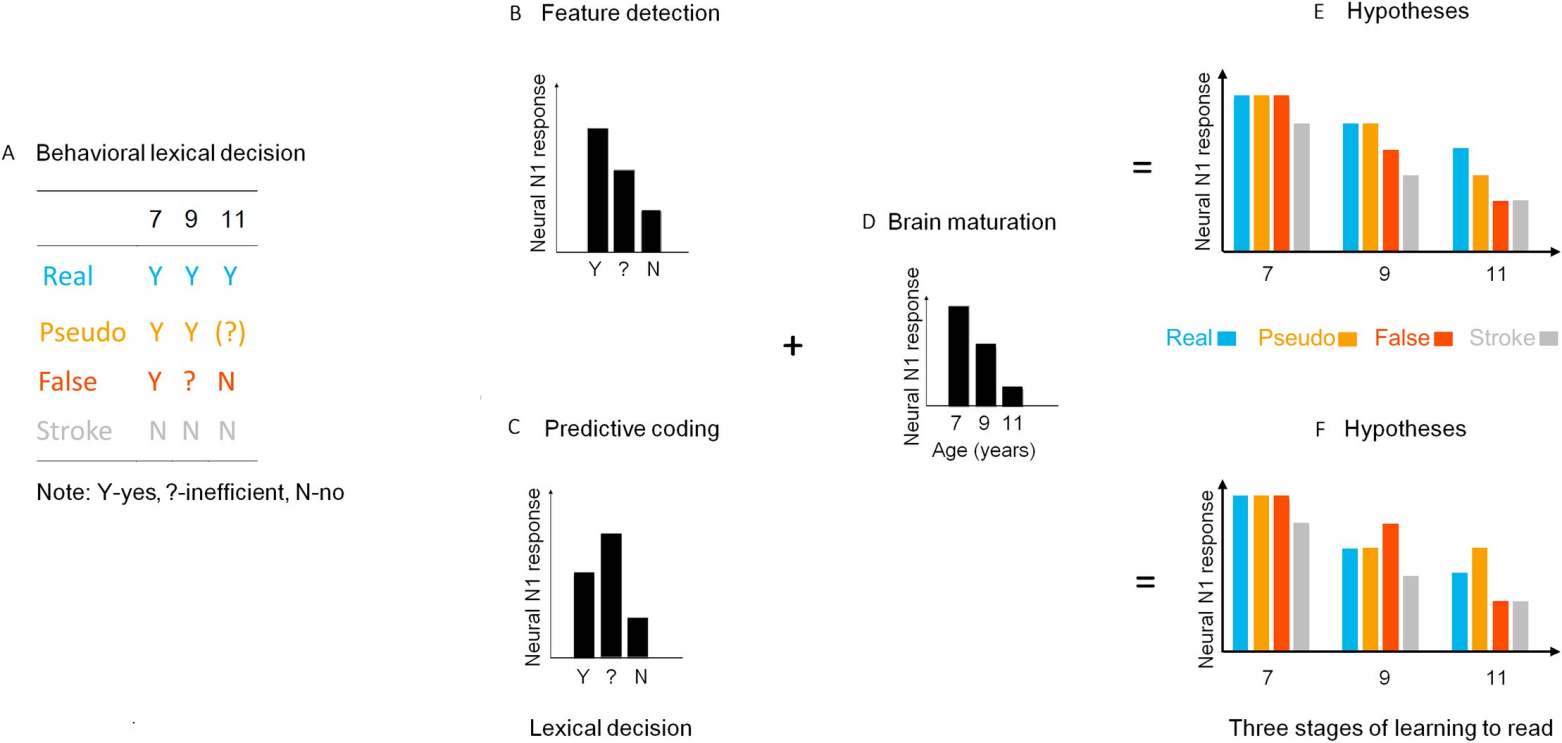
Model specification. (A) Orthographic regularity perception and lexical classification efficiency in children across 3 stages of learning to read (also see Fig 2). Hypothesized neural N1 responses in feature detection model (B) and predictive coding model (C). (D) Neural response of the system with age decrease due to general brain maturation. (E) Hypothesized development of neural responses to stimuli with different orthographic regularities at 3 stages of learning to read based on the feature-detection model. In the beginning readers (7 years old), neural response to real/pseudo/false characters with higher orthographic features would be stronger than that to stroke combinations. In the middle stage (9 years old), the neural response to false characters would be reduced and weaker than that to real/pseudo characters with higher orthographic features. With further reading training, in the later stage (11 years old), neural response to pseudo characters (less regular) would be reduced and weaker than real characters. (F) Hypothesized development for the predictive coding model. In the beginning stage, the neural response to real/pseudo/false characters would be stronger than that to stroke combinations (fewer predictions and prediction errors). In 9-year-olds, neural response to false characters (lower efficiency of prediction due to higher prediction error) would be increased and stronger than that to real/pseudo characters. In 11-year-olds, neural response to pseudo characters (lower efficiency of prediction due to higher prediction errors) would be stronger than that to real and false characters.

Participants’ responses (i.e., character versus non-character; see Fig 2) were analyzed using a generalized linear mixed-effect model using the lme4 package [7] in R [8]. The model evaluated categorical responses as predicted by the main effect of Stimulus Type (real, pseudo, false, strokes), Age (7, 9, 11), and the interaction of the two, including random intercepts by Subjects and Items and slopes of Stimulus Type by Subject (see S1 Table, S2 Table, S3 Table, S4 Table, S5 Table, S6 Table and S1 Text for details). Results show that the main effect of Stimulus Type, *χ*^*2*^(3) = 819.720 (*p <* 0.001), and the main effect of Age, *χ*^*2*^(2) = 11.605 (*p <* 0.01), were significant. Importantly, the interaction between Stimulus Type and Age was significant, *χ*^*2*^(6) = 45.404 (*p <* 0.001). These results suggest that different response patterns of lexical classification manifested in children at different ages. To further link the behavioral response to the neurophysiological N1 amplitude under the framework of predictive coding, we further compared subjects’ percentage of ‘character’ response to chance level, which is taken as a measure of the orthographic uncertainty (in contrast to clear-cut representation) of the different types of stimuli for each group of subjects. For this comparison, we conducted a binomial test (with Bonferroni adjustments for multiple comparisons). The 2 theoretical models of reading make different predictions regarding the comparisons of N1 responses to stimuli with different levels of uncertainty as reflected in the proximity to chance-level lexical classification (see Fig 3). Specifically, if the percentage of character response was high above chance level (50%), participants perceived the stimulus type as having high orthographic regularity and were more efficient in classifying them as real characters (associated with sounds and meanings, i.e., potential sources of prediction), presumably generating lower prediction error. Importantly, if the percentage of character response was close to chance level, participants were more uncertain about these stimuli and were less efficient in lexical classification, presumably generating higher prediction error (a larger difference between visual input and predictions, [4]). If the percentage of character response was lower than chance level, participants were able to more efficiently reject these stimuli as real characters and were less likely to engage the word-processing mechanism, presumably generating lower prediction error. As shown in Fig 2A, we found that across 3 age levels, participants consistently responded ‘character’ to real and pseudo characters and ‘non-character’ to stroke combinations: the percentages of character response to real and pseudo characters were high above chancel level (all *p* < 0.001), and the percentage of character response to stroke combinations was well below chance level (all *p* < 0.001). Interestingly, a developmental change was found in the percentage of character response to false characters: high above chance level at 7 years (*p* < 0.001), close to chance level at 9 years (*p =* 0.136), and then lower than chance level at 11 years (*p =* 0.012). Because the percentage of character responses for real and pseudo characters are near ceiling, we further analyzed participants’ reaction times in classifying whether a stimulus was a real character (i.e., character response). Longer reaction time means that the participants were less efficient in classifying it as a real character, with higher prediction error. For reaction time measures, we focused on the high-frequency Chinese characters. Reaction times were calculated from trials of the character response. Reaction times shorter than 200 ms (0.48% of all trials) were discarded before further statistical analysis. One outlier participant reaction time was replaced by the group mean plus 3 standard deviations. All data were analyzed using SPSS 22.0 (IBM Corp, https://www.ibm.com/analytics/spss-statistics-software). Data were analyzed using the General Linear Model (GLM) procedure for repeated measures to model 2 types of Stimulus (real character and pseudo character), and 3 levels of Age (7, 9, and 11). As shown in Fig 2B, the results revealed no significant Stimulus Type difference: *F*(1, 38) = 0.407 (*p* > 0.05). The Age difference was marginally significant: *F*(2, 38) = 2.611 (*p =* 0.087). Importantly, the effect of Stimulus Type by Age was marginally significant, *F*(2, 38) = 2.597 (*p =* 0.088, power = 0.582). And further, tests of simple effects and Bonferroni-adjusted post hoc comparisons were made using estimated marginal means (EMMEANS) procedure within this model. Results showed a longer reaction time for pseudo characters than real characters in 11-year-olds (*p =* 0.045, Hedges’s *g* = 0.811), but no significant difference was found in either 7- or 9-year-olds (all *p >* 0.05).

As shown schematically in Fig 3A, our first experiment suggests that children gradually acquire knowledge about orthographic regularity of characters and develop in the perception of the orthographic regularity and the efficiency of lexical classification during their reading development: (1) young children with little reading experience perceived real, pseudo, and false characters as having similar high regularity and showed high efficiency in responding (low prediction error); (2) those children with 2 more years of reading training perceived false characters as less regular but were less efficient for false characters than real and pseudo characters in lexical classification (higher prediction error); and (3) those children who had more reading experience more efficiently rejected false characters as real characters (lower prediction error), but perceived pseudo characters as less regular and were less efficient for pseudo characters than real characters in lexical classification (higher prediction error).

### ERP

We next used the ERP method to directly test the 2 theories of reading acquisition described above, addressing the core question: how a child’s brain develops specialization for print. We focused on the N1 (N170) component, which is the first negative component of the ERP occurring at posterior electrodes (and peaking at 170 ms in adults). We focused on this component because existing studies comparing words to low-regularity control stimuli have shown that the neural specialization for print is reliably distinguished as early as 130 to 170 ms over the occipitotemporal cortex, peaking at the level of the N1 (N170) component [1,5,9]. We used a content-irrelevant color-matching task with a short duration of stimulus presentation (Fig 1B) to target nonstrategic automatic predictions, because long stimulus durations and explicit language tasks may lead to confounding effects from task-related linguistic predictions on early visual-orthographic processes [3,10,11]. S7 Table and S2 Text provided empirical evidence that the top-down predictions were nonstrategic and generated automatically, thus precluding task effects that could potentially occur during the N1 time range [10–12].

Based on the first experiment, the 2 models make different predictions regarding the pattern of N1 responses mainly to the 3 types of stimuli that are likely to engage word processing mechanisms but with different orthographic regularity (i.e., real, pseudo, and false characters) (also see Fig 3). In other words, focusing on these 3 types of stimuli allowed for a more sensitive test between the two models. Specifically, the feature detection model proposes that neural N1 responses to print are primarily driven by the perception of orthographic features and hypothesizes that lower orthographic regularity would lead to weaker N1 (Fig 3B). Neural responses would monotonously decrease for stimuli with increasingly lower regularity (false characters and pseudo characters) as children become more skilled in detecting the stimulus orthographic regularity (Fig 3E). To the contrary, the predictive coding model hypothesizes that neural response would be stronger for stimuli with lower regularity when efficiency for the stimuli in lexical classification was reduced (and thus higher prediction error) (Fig 3C, also see [4] for more discussion). Neural N1 responses would show a non-monotonic pattern, with strongest response to false characters at 9 years and pseudo characters at 11 years (Fig 3F). As discussed before, children aged 9 were less efficient in lexically classifying false characters (i.e., more confused about false characters), indicating a higher tendency to engage the word processing mechanism, resulting in high prediction error (due to mismatch between input false characters and top-down prediction), whereas children aged 11 were more efficient in rejecting false characters as real characters thus reducing engagement of the word processing mechanism and allowing less opportunity for prediction error to manifest (also see Fig 2 and Fig 3A). Consequently, based on the predictive coding model that postulates stronger neural response for stimuli with higher prediction error [4], we predicted that N1 response to false characters would be decreased between 9 and 11 years.

**Fig 4:**
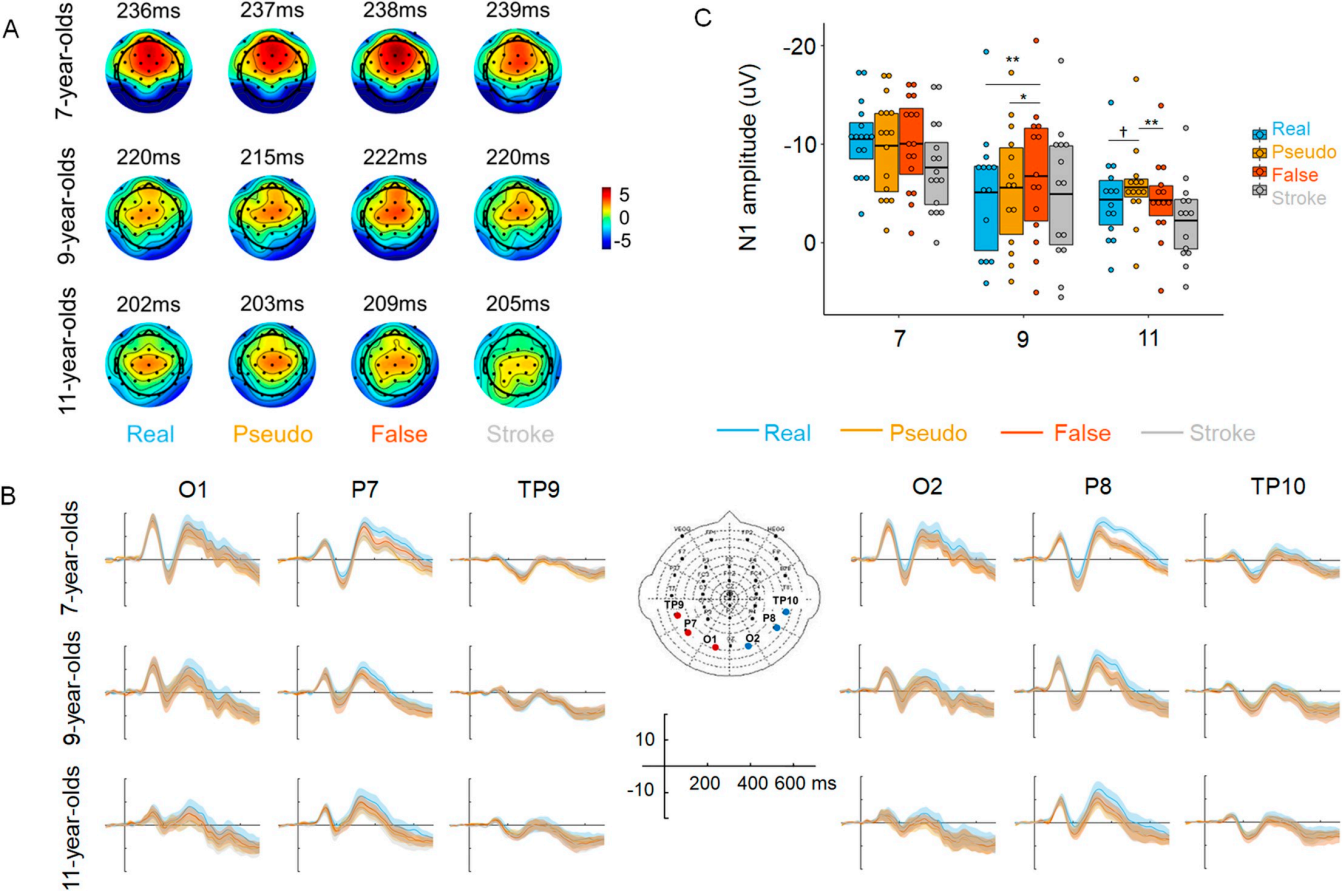
Development of N1 responses to stimuli with different orthographic properties at 3 stages of learning to read. (A) Topographic maps at N1 peaks of 4 types of stimuli on P7 electrode across 3 age groups (see S8 Table and S4 Text for detailed results of N1 peak latency). (B) Grand ERP waveforms of 4 types of stimuli at occipitotemporal electrodes in each group (left panel: O1/P7/TP9 electrodes on the left hemisphere; right panel: O2/P8/TP10 electrodes on the right hemisphere). Error bands are 95% nonparametric CIs (2,000 bootstraps) (C) Box plots of mean N1 amplitudes for the 4 stimulus types at left occipitotemporal electrodes in each group. At the beginning of learning to read (7 years), no N1 difference was observed among the 3 types of stimuli (real, pseudo, and false characters) that were perceived as character-like. After about 2 years of reading training (9 years), N1 responses were observed to be greater for lower orthographic-regularity false characters that were not predicted efficiently (high prediction error) than for real and pseudo characters, whereas no difference was found between the latter two types of stimuli. With further reading training (11 years), pseudo characters with lower efficiency of prediction (higher prediction error) evoked a stronger N1 than false and real characters. Lines in the boxes denote means; **p < 0.01, *p < 0.05, ^†^*p <* 0.1. See 10.6084/m9.figshare.8948912 for subject data. ERP, event-related potential.

Fig 4A illustrates topographic maps elicited by each stimulus type. We found a robust effect of Stimulus Type by Lateralization by Age (*F*[3.87, 79.41] = 4.721; *p =* 0.002; power = 0.99, with Greenhouse Geisser corrected) over the occipitotemporal electrodes. And further, simple effects tests and Bonferroni adjustments for post hoc comparisons were made using EMMEANS procedure within the GLM model. Results of this analysis showed that the N1 amplitudes elicited by the 3 types of orthographic stimuli (real, pseudo, and false characters) are stronger than that for stroke combination (see S3 Text for detailed results).

The critical results regarding our hypothesis were shown in Fig 4C, which illustrates non-monotonic pattern of N1 responses to the 3 types of orthographic stimuli over the left occipitotemporal electrodes across 3 stages of learning (see S3 Text for detailed results). While 7-year-old children showed similar N1 responses to real, pseudo, and false characters, 9-year-old children showed stronger N1 to false characters than real and pseudo characters. In 11-year-old children, N1 to pseudo characters was stronger than false and real characters. Over the right side, no N1 difference was found among the 3 stimulus types and no developmental change in the pattern of N1 responses to these types was shown among 3 age levels.

Taken together, we observed a stronger N1 response to stimuli with lower orthographic regularity (less character-like) but only when efficiency in lexical classifying for these stimuli was reduced (high prediction error) at a certain age, as indicated by stronger N1 responses to false characters in the 9-year-olds and to pseudo characters in the 11-year-olds, respectively. These results unequivocally support a predictive coding account of the development of the neural specialization for print (comparing Fig 4C to Fig 3F) but are incompatible with hypothesized responses based on feature detection models (comparing Fig 4C to Fig 3E). As discussed before, focusing on these 3 types of stimuli allowed for a more sensitive test between the 2 models. That said, results from GLM procedure including all the experimental levels (real, pseudo, false, stroke combination) showed the same overall pattern of results as that of the more constrained GLM procedure, in particular regarding the significant difference between false and pseudo characters at 9 and 11 years (see S3 Text for detailed results).

## Discussion

The goal of this study was to address the question “How do children develop the neural specialization for print through the predictive coding mechanism?” The behavioral data from the lexical decision task with stimuli differing in the degree of orthographic regularity suggest that orthographic regularity is learned over the course of several years of reading acquisition (see [13] for discussion) and that the efficiency at which a certain stimulus type can be classified as real characters changes over time, showing a non-monotonic developmental profile (see Fig 2 and Fig 3A). At the neural level, these learning effects are indicated by changes in N1 amplitude reflecting prediction error: at the beginning of learning to read, children became sensitive to orthographic regularities of how strokes construct radicals but were not sensitive to how radicals are positioned to construct characters. Therefore, the top-down predictions based on classification (lexical decision) do not differentiate between real characters, pseudo characters, and false characters, leading to similar prediction errors indicated by similar N1 responses across these 3 types of stimuli. With further reading training, children start to pick up some orthographic regularities of higher-ordered visual units [13], such as how radicals construct characters (e.g., the position of radicals) in Chinese characters. Before these regularities are well learned, the predictions for these stimulus types were less efficient, reflected in larger prediction errors indicated by larger N1 responses (e.g., false characters at 9 years in our study). With more learning, children became more familiar with the orthographic regularities of how radicals construct characters and were able to more efficiently reject the stimulus types not conformed to the regularity of radical position of Chinese characters (i.e., false characters); they were also less likely to engage top-down prediction, resulting in low prediction errors indicated by lower N1 responses (e.g., false characters at 11 years in our study). They have also acquired more higher-order regularities (i.e., character level such as the combination of the radicals in Chinese, similar to n-gram frequencies in alphabetic words). However, these higher-order orthographic regularities are not well learned yet, leading to inefficient prediction and larger prediction errors and stronger N1 amplitudes (e.g., pseudo characters at 11 years in our study). Taken together, our findings indicate that the neural specialization for print develops from the interaction of stimuli’s orthographic regularity and efficiency of lexical classification that are optimized by reading experience across 3 stages of learning. Yet this explanation still requires more evidence for verification in future studies. By using a larger set of tests that more directly measure the prediction and prediction error and examining their contribution to the development of neural specialization for print in children during learning to read, further results may provide more evidence for clarifying the predictive coding mechanism underlying how the brain learns to read.

As a general principle, predictive coding presumably occurs at multiple levels in the brain and under many circumstances. In the context of reading, specifically based on the Interactive Account advocated by Price and Devlin (2011) [4] to explain how a developing brain acquires specialized visual-orthographic processing, 2 types of top-down predictions are suggested: the task-dependent strategic predictions and the relatively task-independent automatic predictions. Previous studies have shown that neural activation changes with task (task-dependent), consistent with the operations of strategic (or task-dependent) predictions [4,14,15]. Our focus in this study is that the 2 models of reading make different predictions regarding the effect of automatic predictions. Therefore, to minimize the contribution from strategic predictions, we used the content-irrelevant color-matching task in the present study. To distinguish between the theoretical hypotheses of the 2 models, we constructed 4 types of stimuli that varied in orthographic regularity at different levels of representations (see Fig 1A). As shown in Fig 2, the orthographic regularity at different levels is gradually learned over the course of several years of reading acquisition. The Interactive Account suggests that children automatically make predictions about the input word-related stimulus based on their experience with similar stimuli [4].

In addition to experience, top-down prediction can also come from the context (e.g., sentence context, picture context, etc.) in linguistic tasks, which would be in the category of strategic and task-dependent prediction (see the review paper [15]). For example, when the brain expects to see a noun in a specific context (e.g., a sentence), the visual cortical response to a word that is phonologically similar to a noun is weaker [14]. Using single words and a content-irrelevant color-matching task, results in the present study suggest that without a specific context such as a sentence, visual expectations about orthographic regularity can be generated automatically and unconsciously from an individual’s previous experience with similar stimuli. Although it is beyond the scope of the current study, it would be interesting to further investigate the interaction between strategic and automatic predictions, to examine whether the same pattern could be seen under different types of tasks, ideally with a larger sample size.

A critical question further arises about where the predictions come from. In agreement with Price and Devlin (2011) [4], we propose that the predictions are generated by higher-level linguistic mechanisms representing semantic and phonological information: when children learn visual-orthographic regularities through exposure to characters and words during reading, the visual word form is mapped onto linguistic properties. Through repeated co-activations of the visual system and the linguistic network, higher-level language regions learn to predict the visual input during reading acquisition. In addition to the possibility that higher-level brain areas such as linguistic areas generate predictions and feed back to modulate the N1 response, it is possible that the predictions are locally generated in occipitotemporal areas, and stimuli with extremely low orthographic regularity may simply not activate the occipitotemporal areas. While outside the scope of the present study, it is certainly important for future studies to delineate the sources of top-down predictions, including linguistic areas, occipitotemporal areas, and higher-level visual or visuospatial areas [16].

The interactive account based on the predictive coding framework, supported by the present study, also offers elegant explanations for other aspects of reading, such as linking orthographic and higher-level language information. Some cognitive models of reading include an orthographic input lexicon as a specific functional model to represent the abstract visual word form ([15–16], recent findings from [17–18]), while others claim that the abstract representation arises from interactions between visual input and semantic and phonological processing without explicit word form representations [17,18]. At the neural level, one longstanding view is that the brain primarily represents perceptual information of visual words (the perceptual hypothesis [19]; the orthographic coding hypothesis [3]), while the other view is that the system mainly serves as an interface between the visual form and the high-level phonological and semantic representations (the interface hypothesis [20]; the distributed circuit view [21]; the graded hemispheric specialization view [22]). The predictive coding account supported by the present results is in agreement with the latter models and suggests that the link of orthographic features with phonology and semantics is very straightforward because inherently phonological and semantic predictions are an essential part of orthographic processing. These results also have consequences about our understanding of orthographic deficits underlying dyslexia. Some of the most robust processing impairments in dyslexia are hypoactivity in the left inferior occipitotemporal brain region [16]. Our results suggest that these deficits may be explained by a lack of predictions from phonological language areas, which is also agreement with the phonological core deficit of dyslexia [23,24], thereby providing a sparse explanation of neural deficits in dyslexia.

Previous studies have repeatedly shown that predictive coding may underlie object recognition [25], low-level visual processing [26], action planning [27], or psychiatric disorders [28]. The present study supports the presence of the predictive coding mechanisms in visual word recognition (also see [29]) and adds converging evidence that predictive coding may be a general mechanism of information processing in the brain [30,31].

### Conclusion

We observed a robust interaction between a stimulus’ orthographic regularity (bottom-up input) and children’s lexical classification ability (top-down prediction): electroencephalogram (EEG) N1 responses were stronger to lower-regularity stimuli, but only in children who were less efficient in lexically classifying these stimuli (high prediction error). In contrast, N1 responses were reduced to lower-regularity stimuli in children who showed high efficiency of lexical classification (low prediction error). The present findings resolve the controversy in understanding the mechanisms of reading skill acquisition and offer very strong support for predictive coding as the underlying mechanism during the development of neural specialization for print.

## Materials and methods

### Ethics statement

For all children who participated in the present study, written informed consent in accordance with the guidelines and approval of the ethical committee of the Hangzhou Normal University (IRB #20130301h) was obtained from their parents or other legal guardian. The protocols adhered to the Declaration of Helsinki.

### Participants

Forty-six primary school children participated in our study, assigned to one of 3 age groups. Participants all had normal or corrected-to-normal vision and were right-handed. As our goal was to characterize normal development, dyslexic children were excluded. As in previous studies of Chinese developmental dyslexia [32–34], a child was considered dyslexic if the score in a Chinese character recognition test was 1 standard deviation below the average of the same grade. Two participants thus were excluded in the present study. Data of 3 participants (one child in each group) were missing because they quit after the ERP experiment. The demographic characteristics of the 44 participants and their performance on a Chinese character recognition test are listed in Table 1.

**Table 1:** Participants information and their reading performance.

Group	*N* (male)	Age, mean (SD)	Reading score, mean (SD)
**7-year-old**	16 (8)	7.15 (0.47)	25.06 (27.21)
**9-year-old**	14 (7)	9.05 (0.38)	114.57 (12.04)
**11-year-old**	14 (6)	10.98 (0.33)	130.64 (4.41)

Children’s reading was assessed in a Chinese character recognition test [35], which has been widely used for measuring Mandarin-speaking Chinese children for reading ability [36–38]. In this test, children were instructed to read a list of 150 Chinese characters arranged in increasing difficulty (see [35] for a detailed description of this test).

### Material

Four types of stimuli were used: real Chinese characters, pseudo characters, false characters, and stroke combinations. Each type of stimuli consisted of 36 items. They were matched regarding structural complexity (all stimuli were formed by 2 horizontally arranged radicals) and average number of strokes (ranging from 5 to 10 strokes). Half of real characters were at low (1–5 per million) frequency of occurrence, and half of those were at high frequency of occurrence (400–4,500 per million) [39]. Children aged 7 to 11 performed similarly near ceiling in the lexical decision task. We did not measure each subject’s semantic/phonological knowledge of these characters; however, based on general survey data, children in these age groups can read slightly more than half of these characters. The typical position is the only legal position for each radical (see Fig 1A). Pseudo and false characters were built with the same set of radicals, except that both radicals appeared at their legal positions for pseudo characters but at illegal positions for false characters. Between the 2 types of stimuli, the radicals were matched at the individual stimulus level by exchanging the relative positions of the two radicals, which resulted in radicals being at legal or illegal positions. And each stroke combination was composed of 2 horizontally arranged nonexisting radicals (each radical was constructed by recombining the strokes of the radical in false characters).

### Procedure

#### Behavior

Children were tested in a lexical decision task with the same stimulus duration (300 ms) as the EEG experiment. Of a total of 36 non-character items, 12 items were stroke combinations, 12 items were false characters, and 12 items were pseudo characters. There were 12 items in each of the 3 types of non-character stimuli (pseudo character, false character, stroke combination). To balance the prior probability of characters and non-characters, we included 36 real characters as fillers. This is a common practice in lexical decision tasks in which more than one type of non-characters is included [40–42]. All the stimuli were the same as those in the EEG experiment. Children were asked to decide whether a stimulus was a real Chinese character or not. They pressed one button for a real character and one button for a non-character. The assignment to give the answer with the left or right index finger was balanced across subjects.

#### ERP

The ERP experiment consisted of 4 blocks. Each block consisted of 108 trials. The 4 types of stimuli were presented randomly, but an additional rule prohibited 2 successive stimuli to be of the same type. Each stimulus was presented in one of the 3 colors (green, red, or yellow) on a gray background in the center of the screen. Each color appeared the same number of times across stimulus types. The horizontal visual angles of stimuli were 1.97°. Each specific trial was constructed as follows: fixed stimulus duration of 300 ms and an interstimulus interval (ISI), uniformly distributed among 1,450 ms, 1,525 ms, 1,600 ms, 1,675 ms, and 1,750 ms and randomized across trials. Participants were instructed to press a button whenever the color of a stimulus occurred 2 times in a row (i.e., one-back color repetition detection). The assignment to give the answer with the left or right index finger was balanced across subjects. There were 36 items per condition, including a subset of 30 stimuli as non-target and the other 6 stimuli as targets (16.67% of all stimuli). To get enough trials for ERP averaging, all stimuli were presented 3 times. A similar practice was used in previous studies [9,43–51]. Therefore, there were 108 trials (90 non-target trials and 18 target trials) for each stimulus condition. The potential effect of stimulus repetition was thus an important potential question. Importantly, the word N170 repetition effects have been found in studies with consecutive presentation of the same stimulus, and under short ISIs (e.g., 200 ms) [52–54], but not in studies using long ISIs (such as 1,000 ms in [55] and jittered between 1,250 and 1,750 ms in [56]). In the current study, the ISI was long (jittered between 1,450 and 1,750 ms). Moreover, the 4 types of stimuli were presented randomly with a rule prohibiting 2 successive stimuli to be of the same type. Therefore, we believe that our main results are likely not affected by this nonconsecutive repetition with long ISI. Nevertheless, the effect of stimulus repetition is indeed an important question.

#### EEG data acquisition and analysis

EEG signal was recorded from 30 Ag/AgCl electrodes secured in an elastic cap according to the extended international 10–20 system. The EEG data were recorded by using a DC-amplifier system (BrainAmp ExG, Brain Products GmbH, Gilching, Germany). The software used for EEG data recording was BrainVision Recoder (Brain Products GmbH Germany). The electrode at AFz served as ground and one electrode between Cz and CPz electrodes served as an online reference, and the data were offline re-referenced to a common average reference over all electrodes except for electrooculogram (EOG) electrodes. Both vertical and horizontal EOGs were also recorded for eye-blink correction. All electrode impedances were kept below 5 kΩ. EEG and EOG were recorded continuously and were amplified with a band pass from AC 0.1 to 100 Hz at a sampling rate of 1,000 Hz.

Offline data processing involved band-pass filtering (low-pass filtering at 30 Hz and high-pass filtering at 0.1 Hz). Before averaging, each recording epoch was manually scanned for artifacts. Trials were corrected for eye movement artifacts offline using an ICA procedure. Trials were epoched and baseline corrected offline with a 100 ms prestimulus period. The duration of the poststimulus period was 800 ms. For all 4 stimulus types, only non-target trials for which no false positive (target) responses occurred entered the further analyses. Before averaging, artifacts exceeding ±100 μV were automatically rejected. In our study as in many other studies, N170 (N1) was used to index the processing of visual objects, in this case, written words and control stimuli. It is well established that the occipitotemporal electrodes are most representative electrodes for measuring the N1/N170 response [1,6,45–48,51,57–59]. Indeed, as shown in Fig 4A, for each of the 4 types of stimuli (i.e., real character, pseudo character, false character, and stroke combination), the topographic maxima corresponding to N1/N170 was located in the occipitotemporal electrodes. Three pairs of occipitotemporal channels (P7/P8, O1/O2, and TP9/TP10) were thus selected based on this topographic map as well as convention for measuring N1/N170. In addition, the 6 electrodes allowed us to test for the lateralization effect. We used a global field power method to determine the time window of the N1 component in each children’s group (7-year-olds: 192–271 ms; 9-year-olds: 188–252 ms; and 11-year-olds: 181–233 ms; respectively). The peak values of N1 at the 6 occipitotemporal electrodes were detected. In the statistical analysis, the N1 amplitudes of these electrodes were averaged for each hemisphere, respectively (i.e., left hemisphere: P7/O1/TP9 electrodes; right hemisphere: P8/O2/TP10 electrodes). And in the lateralization analysis, the peak values of N1 at these electrodes were averaged for each hemisphere (the left hemisphere: P7/O1/TP9; the right hemisphere: P8/O2/TP10). Peak latencies of N1 were obtained at the most typical P7/P8 channels. Effect sizes of the main comparisons were reported [60–62]. The power of the main results in each experiment was estimated based on the current sample size using the GPower program [63].

## Supporting information

S1 Text Results in the generalized linear mixed-effect model in the lexical decision task. (DOCX)

S2 Text Results of hit rate and reaction time in the color-matching task. (DOCX)

S3 Text Results of the N1 peak amplitude in the color-matching task. (DOCX)

S4 Text Results of the N1 peak latency in the color-matching task. (DOCX)

S1 Table Results of ANOVA (model1, model2). The main effect of stimulus type in the generalized linear mixed-effect model in the lexical decision task.
(DOCX)

S2 Table Results of ANOVA (model3, model2). The main effect of age in the generalized linear mixed-effect model in the lexical decision task.
(DOCX)

S3 Table Results of ANOVA (model2, model4). The interaction of stimulus type by age in the generalized linear mixed-effect model in the lexical decision task.
(DOCX)

S4 Table Results of ANOVA (model4, model5). The random intercept by item in the generalized linear mixed-effect model in the lexical decision task.
(DOCX)

S5 Table Results of ANOVA (model6, model5). The random intercept by subject in the generalized linear mixed-effect model in the lexical decision task.
(DOCX)

S6 Table Results of ANOVA (model5, model7). The random intercept by slopes of stimulus types by subjects in the generalized linear mixed-effect model in the lexical decision task.
(DOCX)

S7 Table Mean hit rate and reaction time in the color matching task. (DOCX)

S8 Table Mean N1 latency of 4 stimulus types at P7/P8 in each group of children. (DOCX)

## Abbreviations

EEG: electroencephalogram
EMMEANS: estimated marginal means
ERP: event-related potential
EOG: electrooculogram
GLM: General Linear Model
ISI: interstimulus interval
N1: the first negative component of the ERP at posterior electrodes
N170: negative component of the ERP peaking at 170 ms at posterior electrodes in adults
