# Supplementary material for: Development of neural specialization for print: Evidence for predictive coding in visual word recognition
Source: PLoS Biol. 2019 Oct 10;17(10):e3000474. doi: 10.1371/journal.pbio.3000474 (PMC6805000; doi:10.1371/journal.pbio.3000474)
Supplement: S1 Text — (DOCX) [file pbio.3000474.s001.docx]

Supplementary Materials for

Development of neural specialization for print: Evidence for predictive coding in visual word recognition

# Results in the generalized linear mixed effect model in the lexical decision task

With the *anova()* function in lme4 package, we examined whether a particular model provides a significantly improved fit to the data over another (i.e. whether one model explained more of the variance in the data than another model). As described below, results showed that the main effect of Stimulus Type, *χ²*(3)= 819.720, *p*<0.001 (see S1 Table) and the main effect of Age, *χ²*(2)= 11.605, *p*<0.01 (see S2 Table) were significant. Moreover, the interaction between stimulus type and age was significant, *χ²*(6)= 45.404, *p*<0.001 (see S3 Table). In addition, the random effect of Item was not significant, *χ²*(1)= 0.176, *p*>0.05 (see S4 Table), while the random effect of Subject, *χ²*(1)= 97.618, *p*<0.001 (see S5 Table) and the random effect of slopes of stimulus types by subjects, *χ²*(9)= 79.910, *p*<0.001 (see S6 Table) were significant.
