# Supplementary material for: Development of neural specialization for print: Evidence for predictive coding in visual word recognition
Source: PLoS Biol. 2019 Oct 10;17(10):e3000474. doi: 10.1371/journal.pbio.3000474 (PMC6805000; doi:10.1371/journal.pbio.3000474)
Supplement: S2 Text — (DOCX) [file pbio.3000474.s002.docx]

Supplementary Materials for

Development of neural specialization for print: Evidence for predictive coding in visual word recognition

# Results of hit rate and reaction time in the color matching task

Hit rates and RTs in the one-back color detection task were analyzed for target trials of the four stimulus types. Three data of participants (two children in 7-year-olds and one child in 11-year-olds) were missing because of file error. The hit rates were calculated by using the number of target trials, which were judged as targets divided by the total number of targets. Means and standard deviations are listed in S7 Table. Data were analyzed using the GLM procedure for repeated measures to model four within-subject levels of Stimulus Type (real, pseudo, false, stroke) and three between-subject levels of Age (7, 9, 11). Greenhouse-Geisser corrections and corrected *F*-values were reported when appropriate. For hit rates, results showed that neither Stimulus Type difference, *F*(2.55, 96.75) = 1.825, *p*>0.05, no the effect of Stimulus Type by Age, *F*(5.09, 96.75) = 0.938, *p*>0.05 was significant. The Age difference was significant, Age, *F*(2, 38) = 5.640, *p*<0.01. Results of the post-hoc comparisons with Bonferroni adjustment using EMMEANs procedure within this model revealed that the hit rate was lower in 7-year-olds than that in 9- and 11-year-olds (*p*<0.05), while no difference was found between 9- and 11-year-olds (*p*>0.05). Reaction times were calculated from hit trials only. In addition, reaction times shorter than 200ms (0.08% of all trials) were discarded before further statistical analysis. Data was analyzed in a similar GLM procedure. The outcomes of this analysis revealed that the Age difference was significant, *F*(2, 38) = 16.330, *p*<0.001. Results of the Bonferroni-adjusted post hoc comparisons using EMMEANs procedure within this model further revealed that the reaction time was longer in 7-year-olds than that in 9- and 11-year-olds (all *p-values*<0.001), while no difference was found between 9- and 11-year-olds (*p*>0.05). However, neither the Stimulus Type difference, *F*(2.94, 111.69) = 0.126, *p*>0.05, nor the effect of Stimulus Type by Age, *F*(5.88, 111.69) = 1.004, *p*>0.05, was significant.

Taken together, the results suggest that children did not show any significant difference in behavioral response to four types of stimuli in the color matching task. In passive viewing and one-back repetition detection tasks in previous studies, participants performed better in response to target letters embedded in native than non-native words or for strings of alphabetic letters than strings of symbols. The increased N1 in response to orthographic stimuli reported in these studies may be partly explained by task-related or strategic modulation. In contrast, we 1) adopted a content-irrelevant color-matching task; 2) found no difference in behavioral performance on the color task across the four stimulus types in each children group. Hence, possible effect caused by task-related or strategic top-down modulation was minimized, and the nonmonotonic N1 effect was more likely due to non-strategic predictions.
