# Supplementary material for: Development of neural specialization for print: Evidence for predictive coding in visual word recognition
Source: PLoS Biol. 2019 Oct 10;17(10):e3000474. doi: 10.1371/journal.pbio.3000474 (PMC6805000; doi:10.1371/journal.pbio.3000474)
Supplement: S3 Text — (DOCX) [file pbio.3000474.s003.docx]

Supplementary Materials for

Development of neural specialization for print: Evidence for predictive coding in visual word recognition

# Results of N1 peak amplitude in the color matching task

Data were analyzed using the GLM procedure for repeated measures to model four with-subject levels of Stimulus Type (real character, pseudo character, false character, stroke combination), two within-subject levels of Laterality (left and right), and three between-subject levels of Age (7, 9, 11). Greenhouse-Geisser corrections and corrected *F*-values were reported when appropriate. The outcome of this analysis revealed significant effects of Age, *F* (2, 41) = 3.732, *p*<0.05, Stimulus Type, *F* (2.56, 105.02) = 28.756, *p*<0.001 and Laterality, *F* (1, 41) = 4.427, *p*<0.05. The effect of Stimulus Type by Age was significant, *F* (5.12, 105.02) = 3.055, *p*<0.05. Neither the effect of Lateralization by Stimulus Type, *F* (2.77, 113.41) = 1.334, *p*>0.05 nor Lateralization by Age, *F* (2, 41) = 0.548, *p*>0.05 was significant. Importantly, the effect of Stimulus Type by Lateralization by Age was significant, *F* (5.53, 113.41) = 3.947, *p*=0.002, *power*=0.99. And further, simple effects tests and Bonferroni-adjusted post hoc comparisons were made using EMMEANs procedure within this model. Results showed that for all groups of children, orthographic stimuli evoked a trend of larger N1 than stroke combination. In the left side, N1 differences between real/pseudo-/false characters and stroke combinations were significant (all *p-values*<0.05) in all cases except for two comparisons in 9-year-olds (real/pseudo characters vs. stroke combinations, *p*>0.05). No N1 difference was found among real, pseudo, false characters in 7-year-olds (all *p*-values>0.05). However, in 9-year-olds, N1 responses were observed to be greater for false characters than for real characters (*p*=0.018) and for pseudo-characters (*p=*0.046), while no difference was found between real and pseudo characters (*p*>0.05). In 11-year-olds, we observed that pseudo characters evoked larger N1 amplitude than false characters (*p*=0.018), while no difference was found between pseudo and real characters or between real and false characters (all *p*-values>0.05). In the right side, differences between real/pseudo/false characters and stroke combinations were significant in all cases (all *p-values*<0.05) except for 9-year-olds (real/pseudo/false characters vs. stroke combinations, *p*>0.05, pseudo/false characters vs. strokes, uncorrected *p*<0.05) or 11-year-olds (real/pseudo characters vs. stroke combinations, *p*>0.05, uncorrected *p*-values<0.05). No N1 difference was found among real, pseudo, false characters in each group (all *p*-values>0.05).

As noted before, the two models make different predictions regarding the pattern of N1 responses mainly to the three types of stimuli with different orthographic regularity (i.e., real, pseudo, false character). To examine these predictions and distinguish the two models, we further focused on the N1 responses to the three types of orthographic stimuli. Data were analyzed using the GLM procedure for repeated measures to model three within-subject levels of Stimulus Type (real, pseudo, false character), two within-subject levels of Lateralization (left and right), and three between-subject levels of Age (7, 9, 11). Greenhouse-Geisser corrections and corrected *F*-values were reported when appropriate. The outcome of this analysis revealed significant Lateralization difference, *F* (1, 41) = 5.036, *p*<0.05 and Age difference, *F* (2, 41) = 4.191, *p*<0.05. Neither the Stimulus Type difference, *F* (1.77, 72.38) = 1.437, *p*>0.05 nor the effect of Stimulus Type by Age, *F* (3.53, 72.38) = 2.077, *p*>0.05*,* The effects of Stimulus Type by Lateralization, *F* (1.94, 79.41) = 1.125, *p*>0.05, Lateralization by Age, *F*(2, 41)=0.357, *p*>0.05 was significant. Importantly, the effect of Stimulus Type by Lateralization by Age was significant, *F* (3.87, 79.41) = 4.721, *p*=0.002, *power*=0.99. To characterize the developmental profile of N1 responses to characters with different orthographic regularity and directly examine the hypothesis of the two models, simple effects tests and Bonferroni-adjusted post hoc comparisons were made using EMMEANs procedure within this model. Specifically, results showed that no N1 difference was observed among the three types of characters (real, pseudo, false characters) in 7-year-olds (all *p*-values>0.05). However, N1 responses were observed to be greater for false characters than for real characters (*p*=0.009, *Hedges’s g*=1.526) and for pseudo characters (*p=*0.023, *Hedges’s g*=1.083) in 9-year-olds. No N1 difference was found between real and pseudo characters (*p*>0.05). With further reading experience, we observed that pseudo characters evoked larger N1 amplitude than false characters (*p*=0.009, *Hedges’s g*=1.218) and real characters (*p=*0.094, *Hedges’s g*=1.176) in 11-year-olds. No N1 difference was found between real and false characters (*p*>0.05). Over the right side, no such N1 difference was found among the three stimulus types in any of the age groups (all *p*-values>0.05).
