# Supplementary material for: Development of neural specialization for print: Evidence for predictive coding in visual word recognition
Source: PLoS Biol. 2019 Oct 10;17(10):e3000474. doi: 10.1371/journal.pbio.3000474 (PMC6805000; doi:10.1371/journal.pbio.3000474)
Supplement: S4 Text — (DOCX) [file pbio.3000474.s004.docx]

Supplementary Materials for

Development of neural specialization for print: Evidence for predictive coding in visual word recognition

# Results of N1 peak latency in the color matching task

S8 Table shows the N1 peak latency of four types of stimuli in children at different ages. Data were analyzed using the GLM procedure for repeated measures to model four within-subject levels of Stimulus Type (real character, pseudo- character, false character, stroke combination), two within-subject levels of Laterality (left and right), and three between-subject levels of Age (7, 9, 11). Greenhouse-Geisser corrections and corrected *F*-values were reported when appropriate. The outcome of this analysis revealed a significant Age difference, *F* (2, 41) = 13.755, *p*<0.001, and Stimulus Type difference, *F* (2.73, 111.73) = 3.481, *p*<0.05. Neither the Lateralization difference, *F* (1, 41) = 0.316, *p*>0.05, nor the effect of Stimulus Type by Age, *F* (5.45, 111.73) = 0.433, *p*>0.05, Lateralization by Age, *F* (2, 41) = 1.082, *p*>0.05, Lateralization by Stimulus Type, *F* (2.71, 111.10) = 0.882, *p*>0.05, Stimulus Type by Lateralization by Age, *F* (5.42, 111.10) = 1.649, *p*>0.05, was significant. Results in the Bonferroni-adjusted post hoc comparisons using EMMEANs procedure within this model showed that N1 latency in 7-year-olds was longer than that in 9- and 11-year-olds (all *p-values*<0.01), while no difference was found between 9- and 11-year-old (*p*>0.05). The latency of pseudo characters was shorter than that of false characters and stroke combinations (all *p-values*<0.05). No difference was found in the other comparisons (all *p-values*>0.05).
