# Supplementary material for: Development of neural specialization for print: Evidence for predictive coding in visual word recognition
Source: PLoS Biol. 2019 Oct 10;17(10):e3000474. doi: 10.1371/journal.pbio.3000474 (PMC6805000; doi:10.1371/journal.pbio.3000474)
Supplement: S1 Table — The main effect of stimulus type in the generalized linear mixed-effect model in the lexical decision task. (DOCX) [file pbio.3000474.s005.docx]

**S1 Table.** Results of anova (model1, model2)

|  | *df* | *AIC* | *BIC* | *logLik* | *Chisq* | *Chi* | *df* | *Pr(>Chisq)* |
| --- | --- | --- | --- | --- | --- | --- | --- | --- |
| model 1^a^ | 4 | 3228.7 | 3252.5 | -1610.4 | 3220.7 |  |  |  |
| model 2^b^ | 7 | 2415.0 | 2456.6 | -1200.5 | 2401.0 | 819.720 | 3 | < 2.2e-16^***^ |

^a^ model 1: accr ~ age + (1 | subj)

^b^ model 2: accr ~ age + type + (1 | subj)
