# Supplementary material for: Development of neural specialization for print: Evidence for predictive coding in visual word recognition
Source: PLoS Biol. 2019 Oct 10;17(10):e3000474. doi: 10.1371/journal.pbio.3000474 (PMC6805000; doi:10.1371/journal.pbio.3000474)
Supplement: S2 Table — The main effect of age in the generalized linear mixed-effect model in the lexical decision task. (DOCX) [file pbio.3000474.s006.docx]

**S2 Table.** Results of anova (model3, model2)

|  | *df* | *AIC* | *BIC* | *logLik* | *Chisq* | *Chi* | *df* | *Pr(>Chisq)* |
| --- | --- | --- | --- | --- | --- | --- | --- | --- |
| model 3^a^ | 5 | 2422.6 | 2452.3 | -1206.3 | 2412.6 |  |  |  |
| model 2^b^ | 7 | 2415.0 | 2456.6 | -1200.5 | 2401.0 | 11.605 | 2 | 0.00302^**^ |

^a^ model 3: accr ~ type + (1 | subj)

^b^ model 2: accr ~ type + age + (1 | subj)
