# Supplementary material for: Development of neural specialization for print: Evidence for predictive coding in visual word recognition
Source: PLoS Biol. 2019 Oct 10;17(10):e3000474. doi: 10.1371/journal.pbio.3000474 (PMC6805000; doi:10.1371/journal.pbio.3000474)
Supplement: S3 Table — The interaction of stimulus type by age in the generalized linear mixed-effect model in the lexical decision task. (DOCX) [file pbio.3000474.s007.docx]

**S3 Table.** Results of anova (model2, model4)

|  | *df* | *AIC* | *BIC* | *logLik* | *Chisq* | *Chi* | *df* | *Pr(>Chisq)* |
| --- | --- | --- | --- | --- | --- | --- | --- | --- |
| model 2^a^ | 7 | 2415.0 | 2456.6 | -1200.5 | 2401.0 |  |  |  |
| model 4^b^ | 13 | 2381.6 | 2458.9 | -1177.8 | 2355.6 | 45.404 | 6 | 3.89e-08^***^ |

^a^ model 2: accr ~ type + age + (1 | subj)

^b^ model 4: accr ~ type * age + (1 | subj)
