# Supplementary material for: Development of neural specialization for print: Evidence for predictive coding in visual word recognition
Source: PLoS Biol. 2019 Oct 10;17(10):e3000474. doi: 10.1371/journal.pbio.3000474 (PMC6805000; doi:10.1371/journal.pbio.3000474)
Supplement: S4 Table — The random intercept by item in the generalized linear mixed-effect model in the lexical decision task. (DOCX) [file pbio.3000474.s008.docx]

**S4 Table.** Results of anova (model4, model5)

|  | *df* | *AIC* | *BIC* | *logLik* | *Chisq* | *Chi* | *df* | *Pr(>Chisq)* |
| --- | --- | --- | --- | --- | --- | --- | --- | --- |
| model 4^a^ | 13 | 2381.6 | 2458.9 | -1177.8 | 2355.6 |  |  |  |
| model 5^b^ | 14 | 2383.4 | 2466.7 | -1177.7 | 2355.4 | 0.176 | 1 | 0.6746 |

^a^ model 4: accr ~ type * age + (1 | subj)

^b^ model 5: accr ~ type * age + (1 | subj) + (1 | item)
