# Supplementary material for: Development of neural specialization for print: Evidence for predictive coding in visual word recognition
Source: PLoS Biol. 2019 Oct 10;17(10):e3000474. doi: 10.1371/journal.pbio.3000474 (PMC6805000; doi:10.1371/journal.pbio.3000474)
Supplement: S5 Table — The random intercept by subject in the generalized linear mixed-effect model in the lexical decision task. (DOCX) [file pbio.3000474.s009.docx]

**S5 Table.** Results of anova (model6, model5)

|  | *df* | *AIC* | *BIC* | *logLik* | *Chisq* | *Chi* | *df* | *Pr(>Chisq)* |
| --- | --- | --- | --- | --- | --- | --- | --- | --- |
| model 6^a^ | 13 | 2479.1 | 2556.3 | -1226.5 | 2453.1 |  |  |  |
| model 5^b^ | 14 | 2383.4 | 2466.7 | -1177.7 | 2355.4 | 97.618 | 1 | < 2.2e-16^***^ |

^a^ model 6: accr ~ type * age + (1 | item)

^b^ model 5: accr ~ type * age + (1 | item) + (1 | subj)
