# Supplementary material for: Development of neural specialization for print: Evidence for predictive coding in visual word recognition
Source: PLoS Biol. 2019 Oct 10;17(10):e3000474. doi: 10.1371/journal.pbio.3000474 (PMC6805000; doi:10.1371/journal.pbio.3000474)
Supplement: S7 Table — (DOCX) [file pbio.3000474.s011.docx]

**S7 Table.** Mean hit rate and reaction time in the color matching task

|  | Hit rates | | | | Reaction Time (msec) | | | |
| --- | --- | --- | --- | --- | --- | --- | --- | --- |
| Age | Real | Pseudo | False | Stroke | Real | Pseudo | False | Stroke |
| 7 | 0.74 (0.04) | 0.73 (0.04) | 0.75 (0.04) | 0.77 (0.04) | 868 (38) | 841 (41) | 838 (36) | 851 (38) |
| 9 | 0.87 (0.04) | 0.90 (0.04) | 0.87 (0.04) | 0.90 (0.04) | 627 (38) | 629 (41) | 651 (36) | 655 (38) |
| 11 | 0.86 (0.04) | 0.89 (0.05) | 0.94 (0.05) | 0.92 (0.04) | 575 (39) | 594 (42) | 561 (37) | 568 (39) |
