# Supplementary material for: Development of neural specialization for print: Evidence for predictive coding in visual word recognition
Source: PLoS Biol. 2019 Oct 10;17(10):e3000474. doi: 10.1371/journal.pbio.3000474 (PMC6805000; doi:10.1371/journal.pbio.3000474)
Supplement: S8 Table — (DOCX) [file pbio.3000474.s012.docx]

**S8 Table.** Mean N1 latency of four stimulus types at P7/P8 in each group of children

|  | P7 electrode | | | | P8 electrode | | | |
| --- | --- | --- | --- | --- | --- | --- | --- | --- |
| Age | Real | Pseudo | False | Stroke | Real | Pseudo | False | Stroke |
| 7 | 236 (5) | 237 (5) | 238 (5) | 239 (5) | 235 (5) | 231 (5) | 238 (5) | 232 (4) |
| 9 | 220 (5) | 215 (5) | 222 (5) | 220 (5) | 214 (5) | 212 (5) | 213 (5) | 219 (5) |
| 11 | 202 (5) | 203 (5) | 209 (5) | 205 (5) | 209 (5) | 207 (5) | 207 (5) | 210 (5) |
